# Obesity, metabolic syndrome, and primary hypertension

**DOI:** 10.1007/s00467-020-04579-3

**Published:** 2020-05-09

**Authors:** Mieczysław Litwin, Zbigniew Kułaga

**Affiliations:** 1 Department of Nephrology and Arterial Hypertension, The Children’s Memorial Health Institute, Warsaw, Poland; 2 Department of Public Health, The Children’s Memorial Health Institute, Warsaw, Poland

**Keywords:** Adolescence, Primary hypertension, Metabolic syndrome, Obesity-related hypertension, Metabolically healthy obesity, Metabolically unhealthy obesity

## Abstract

Primary hypertension is the dominant form of arterial hypertension in adolescents. Disturbed body composition with, among other things, increased visceral fat deposition, accelerated biological maturation, metabolic abnormalities typical for metabolic syndrome, and increased adrenergic drive constitutes the intermediary phenotype of primary hypertension. Metabolic syndrome is observed in 15–20% of adolescents with primary hypertension. These features are also typical of obesity-related hypertension. Metabolic abnormalities and metabolic syndrome are closely associated with both the severity of hypertension and the risk of target organ damage. However, even though increased body mass index is the main determinant of blood pressure in the general population, not every hypertensive adolescent is obese and not every obese patient suffers from hypertension or metabolic abnormalities typical for metabolic syndrome. Thus, the concepts of metabolically healthy obesity, normal weight metabolically unhealthy, and metabolically unhealthy obese phenotypes have been developed. The risk of hypertension and hypertensive target organ damage increases with exposure to metabolic risk factors which are determined by disturbed body composition and visceral obesity. Due to the fact that both primary hypertension and obesity-related hypertension present similar pathogenesis, the principles of treatment are the same and are focused not only on lowering blood pressure, but also on normalizing body composition and metabolic abnormalities.

## Epidemiology end aetiology of arterial hypertension in children and adolescents

**Fig. 1:**
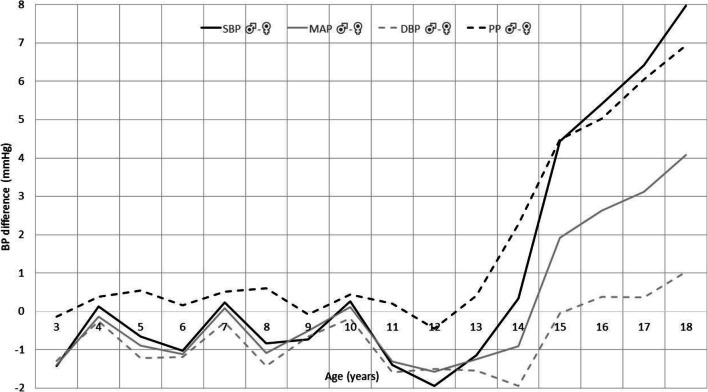
Blood pressure differences between boys and girls 3–18 years of age. *N* = 21,332 (based on results of OLAF Study Poland [7], unpublished data); DBP, diastolic blood pressure; MAP, mean arterial pressure; PP, pulse pressure; SBP, systolic blood pressure

Arterial hypertension (AH) is one of the most important problems of public health worldwide and the main, potentially reversible, cause of cardiovascular disease (CVD). It is estimated that the prevalence of AH accounts for 3–5% in children and adolescents aged 0 to 18 years with much higher prevalence starting from puberty and reaching 10–11% at age 18, which is similar to the prevalence of AH among adults aged 18–45 (10–15%) [1–5]. Moreover, prevalence of elevated/high-normal blood pressure among youth in the USA was found to be 12% [6]. A rapid increase in the prevalence of AH observed during puberty together with growth spurt is seen mainly in boys and is related to the physiological increase of systolic blood pressure (SBP) seen especially in boys (Fig. 1). This sex-related difference in SBP increase corresponds with a greater ratio of boys to girls (3–4:1) among adolescents with primary hypertension (PH) [8]. Historically, the most prevalent form of AH in childhood is used to be secondary AH; however, the situation has changed within the last two decades, and PH is becoming the dominant cause of AH in children above 6 years of age, especially in adolescents [9].

## Determinants of blood pressure

**Fig. 2:**
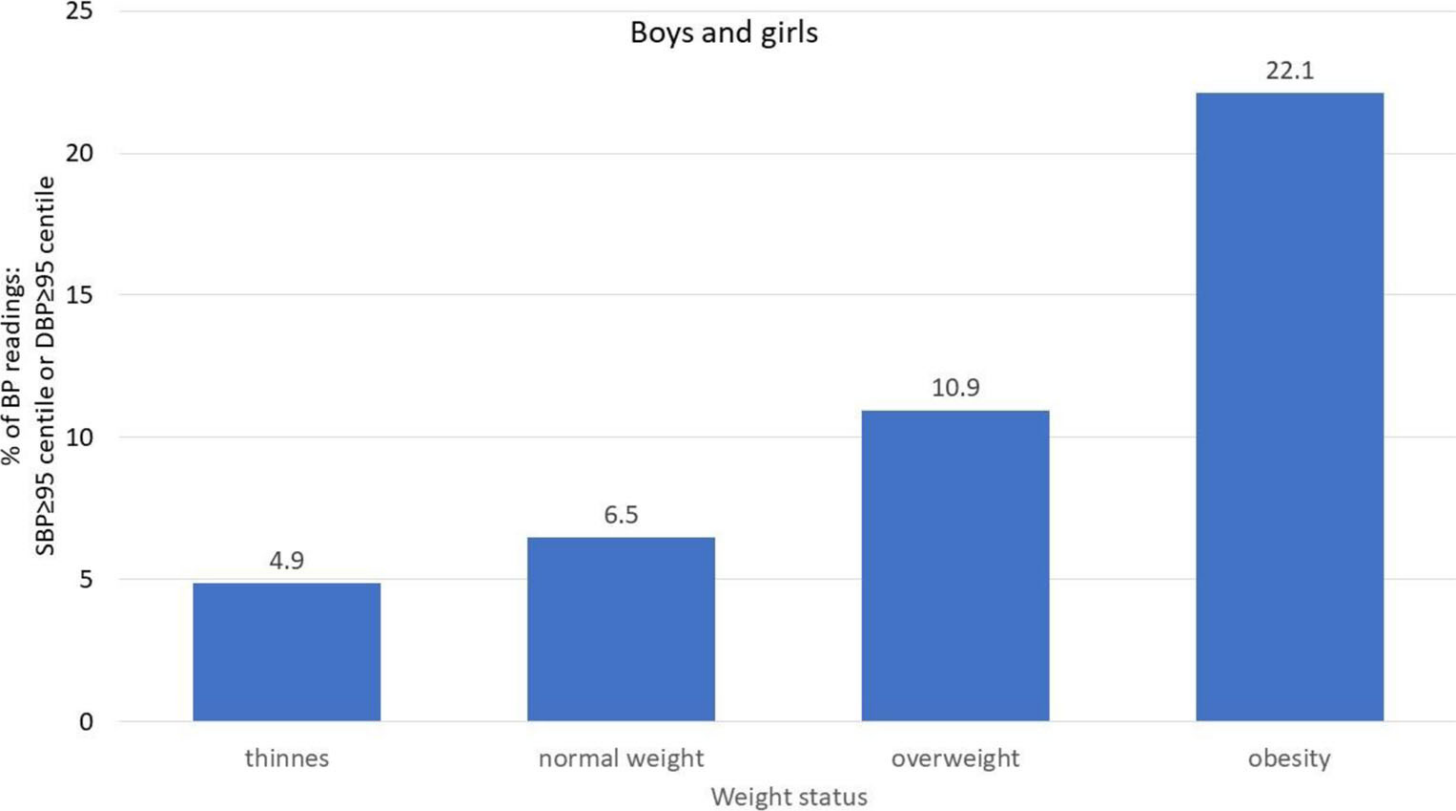
Distribution of blood pressure readings above 95th percentile for age and height according to weight status in 3–18 years old children and adolescents. *N* = 21,404 (based on results of OLAF Study Poland [7], unpublished data)

Blood pressure is regulated by several neurohormonal systems responsible for ensuring proper tissue and organ perfusion. However, the main determinants of the values of population blood pressure are body mass index (BMI) and body composition, specifically visceral obesity and the relations between lean body mass (muscles) and the amount of adipose tissue. The impact of BMI and changed body composition on blood pressure also mediates the impact of socioeconomic status (SES), birth weight, and other CVD risk factors [10]. In epidemiological studies, it has been observed that BMI and other markers of fatness help to identify children with elevated blood pressure [2, 11]. In a cross-sectional, randomized population study from Poland, it was observed that there was a significant increase in the prevalence of high blood pressure values across the BMI categories from underweight to obese (Fig. 2). Similarly, in a study conducted in Canada, it was observed that even after the adjustment to serum fasting insulin levels, heart rate (a marker of the sympathetic drive), and parental history of hypertension, there was a significant increase in SBP with an increased quintile of BMI, both in girls and in boys. The effect was demonstrated in children and adolescents aged 9, 13, and 16 [12]. Moreover, in the general population, weight changes, expressed as weight z-scores, from birth and in any period afterwards, and through childhood to adolescence, significantly affect blood pressure, and the recent weight z-score is related to blood pressure to a larger extent than weight in the past [13]. The same effect was found for subscapular and triceps skinfold thickness. In another population-based study conducted in adolescent girls and boys, each increase in the BMI unit, by 1 cm in waist circumference (WC) and 1 mm in the thickness of triceps subscapular skinfold, was associated with an increase of SBP by 0.7, 0.24, and 0.4 mmHg, respectively [14]. The relation between BMI and blood pressure in childhood and adolescence was recently analyzed in a large study consisting of seven cross-sectional surveys conducted in China, Korea, Poland, the USA, India, Iran, and Tunisia [15]. It was observed that the relationship between BMI and elevated blood pressure had its beginning already at the 25th BMI percentile. These relationships are especially important since in the last few decades, the prevalence of overweight and obesity among children and adolescents has increased worldwide and it is estimated that it exceeds 20%—and in some countries, even 30% [7, 16–19]. Important to note is that in the USA, according to data from NHANES reports 2003–2004 and 2009–2010, 18.8% of children and adolescents age 2 to 18 years were viscerally obese [20]. However, because not all children with PH are obese and not all obese children suffer from PH, the question is being considered whether PH and obesity-related hypertension are the same disease.

## Prevalence of hypertension among obese children

According to a recent Mortality and Morbidity Weekly Report, 2 to 14% of adolescents aged 12–19 years in the USA suffer from elevated blood pressure [6]. Nevertheless, the prevalence of PH is related to weight status. Despite the significant increase in the prevalence of obesity, the prevalence of PH declined between the years 2001 and 2016, even among overweight adolescents, but it increased significantly among obese and severely obese patients. In an analysis of data from over 57,000 overweight and obese children from Germany, Austria, and Switzerland, it was found that, depending on blood pressure reference values, PH was found in 20 to over 40% of subjects [21]. In another analysis of the same group, which included data from 63,025 overweight and obese children and adolescents, it was found that PH was the main comorbidity in the case of overweight and obesity [22]. The prevalence of PH increased from 5% among normal weight children to 20% among overweight children, 26% among obese children, and 39% among children suffering from severe obesity. The blood pressure status was classified according to the Fourth Task Force Report, and stage 1 dominated, but the prevalence of stage 2 also increased with the increase in the BMI status, from 0.4% in normal weight patients to 15.3% among patients with severe obesity. Although blood pressure increased with BMI status in both boys and girls, the absolute blood pressure values were higher among boys than among girls. The second finding of this large study was that another comorbidity related to an increased BMI was dyslipidaemia, including elevated total cholesterol, low HDL cholesterol, high LDL cholesterol, and high triglyceride levels. Although serum insulin was not determined in this study, it is known that hyperinsulinaemia accompanied by inflammation in insulin-sensitive tissues and insulin resistance (IR) are the main metabolic abnormalities in obese patients [23]. Nevertheless, hyperinsulinaemia and IR are not associated with the amount of adipose tissue, but with its distribution. It is the visceral adipose tissue (VAT) that determines IR, and the subcutaneous adipose tissue (SAT) may play a protective role.

In healthy humans, hyperinsulinaemia increases the sympathetic adrenergic drive, but it is offset by a decrease in peripheral vascular resistance. However, chronic hyperinsulinaemia may not decrease peripheral vascular resistance to the same extent. Data from experimental and human studies has shown that increased sympathetic activity, caused by chronic hyperinsulinaemia and inflammation, promotes IR and increases peripheral vascular resistance [24]. Thus, it suggests that sympathetic activation plays a role in the elevation of blood pressure in obese patients. This view is supported by observations of patients suffering from rare, genetic forms of severe obesity. In patients with leptin deficiency and patients with the most common genetically determined form of obesity, i.e., melanocortin-4 receptor mutation, all metabolic complications of morbid obesity are developed, but they are not accompanied by AH and signs of sympathetic activation [24, 25]. These mutations lead to the disruption of signal transduction in the central nervous system and lack of sympathetic activation.

## What do children and adolescents with primary hypertension look like?

The typical phenotypic features of children and adolescents with PH are presented in Table 1. The phenotype indicates that PH is not only a haemodynamic phenomenon, but also a syndrome of interrelated neuro-immuno-metabolic abnormalities leading to haemodynamic consequences [26].

**Table 1:** Common features of intermediate phenotype of primary hypertension and obesity-related hypertension

Features of phenotype of primary hypertension and of obesity-related hypertension	
•Decreased ratio of lean mass/body weight	
•Visceral obesity	
•Accelerated biological maturation	
•Metabolic abnormalities typical of metabolic syndrome—NWMU or MUHO phenotype	
•Oxidative stress	
•Sympathetic nervous system activation	
•Features of accelerated arterial ageing: increased carotid IMT, increased arterial stiffness (PWV), decreased FMD	
•Immune activation—activation of innate and adaptive immunity: more mature/memory/senescent T cells	

### Body composition

In a cross-sectional study conducted in the USA, Flynn and Alderman reported that prehypertensive children were younger, their BMI values were lower, and the prevalence of obesity among them was 20%, while hypertensive children were older, their BMI was within the range of overweight, and the prevalence of obesity among them was 50% [27]. In a recent study, it was observed that BMI values increased with an increase in blood pressure status from below the 80th percentile, 80–90 to the > 90th percentile [28]. However, average BMI in adolescents with blood pressure above the 90th percentile was at the 86th percentile. Similarly, in our study, 29% of adolescents with PH were obese and 53% were overweight [29]. Analysis of body composition with the use of dual X-ray densitometry (DXA) showed that the relation between adipose mass and lean body mass is disturbed in hypertensive adolescents [30]. Chen et al., who analyzed body composition in a population sample of Chinese children and adolescents, observed that metabolic abnormalities typical for metabolic syndrome (Table 2) were present in both obese and normal weight children and were associated with VAT rather than BMI [32]. Secondly, blood pressure was similar in both normal weight children with metabolic abnormalities typical of metabolic syndrome (normal weight metabolically unhealthy [NWMU]) and obese children with metabolic abnormalities (metabolically unhealthy obese [MUHO]), but it was significantly higher than in children with normal body weight who did not suffer from any metabolic abnormalities (normal weight metabolically healthy [NWMH]) and in obese children without metabolic abnormalities (metabolically healthy obese [MHO]). These studies indicate that it is not only adiposity, but disturbed relations between fat mass and lean mass and fat distribution with visceral fatness and metabolic abnormalities that is typical for PH [30, 32–35]. It also means that even in patients with normal BMI but lower lean mass and relatively greater amount of fat mass with visceral distribution (NWMU), blood pressure was elevated and was accompanied by typical metabolic abnormalities [34].

**Table 2:** Paediatric definition of metabolic syndrome according to the International Diabetes Federation [31]

Age (years)	Criteria
< 10	Cannot be diagnosed but further investigations should be done if positive family history towards type 2 diabetes mellitus, cardiovascular disease, and dyslipidaemia
10–< 16	Obligatory criterion: waist circumference ≥ 90 cc or adult cutoff if lower plus 2 or more of the following criteria: Triglycerides ≥ 150 mg/dl HDL cholesterol < 40 mg/dl SBP ≥ 130 and/or DBP ≥ 85 mmHg and/or antihypertensive treatment Fasting glucose ≥ 100 mg/dl or T2DM
≥ 16	Adult criteria: Obligatory criterion: waist circumference ≥ 94 cm for men and ≥ 80 cm for women, plus 2 or more of the following criteria: HDL cholesterol < 40 mg/dl in males and < 50 mg/dl in females Triglycerides ≥ 150 mg/dl SBP ≥ 130 and/or DBP ≥ 85 mmHg, or antihypertensive treatment Fasting glucose ≥ 100 mg/dl or T2DM

### Metabolic abnormalities and metabolic syndrome in adolescents with PH

The role of obesity and visceral fatness as CVD risk factors has been known since the first reports from the Framingham study. However, the exact role of metabolic abnormalities associated with visceral fatness was first described in 1987 by Reaven and Hoffman and then in 1988 by Reaven, who found that hyperinsulinaemia, IR, with accompanying dyslipidaemia, was a consequence of visceral obesity and played a role in the pathogenesis of PH [35, 36]. They also observed that simple reduction of BP did not reduce IR and cardiovascular risk. Now, this cluster of metabolic, anthropometric, and haemodynamic abnormalities is known as metabolic syndrome. The main problem with the formulation of a paediatric definition of metabolic syndrome is that insulin sensitivity, serum lipid concentrations, and anthropometrical variables change with age, and at least 40 different definitions of metabolic syndrome in children have been used [37]. In 2007, the International Diabetes Federation (IDF) published a definition and criteria of metabolic syndrome in children and adolescents [38]. According to the IDF definition, metabolic syndrome can be diagnosed in children older than 10, but not younger; however, children below 10 who meet the criteria of metabolic syndrome should be treated as a risk group. The main and obligatory criterion of metabolic syndrome in the IDF definition is an increased WC, which is a surrogate marker of VAT. Thus, this definition may also include patients with normal BMI. Currently, the definition of metabolic syndrome in children and adolescents used most frequently is the IDF definition, used mainly in Europe, and the definition by Cook et al. is used in the USA (Table 2) [31].

The relationship between BMI/WC, insulinaemia, and blood pressure was observed already in children at the age of 4, in whom serum insulin concentrations, glycaemia, and IR rose with increasing blood pressure status from normotension through prehypertension to AH [39]. The adiposity measures and metabolic abnormalities were higher in hypertensive patients than in normotensive patients. With age, at least until young adulthood (19–42 years), these abnormalities progressed in prehypertensive and hypertensive patients. Hyperinsulinaemia and IR seem to precede the development of PH. Sinaiko et al. reported that higher insulin levels and IR at the age of 13 predicted both the elevation of blood pressure and the development of dyslipidaemia at the age of 16, independently of BMI [40]. Other abnormalities typical of metabolic syndrome, i.e., low HDL cholesterol and high triglycerides, are surrogate markers of IR and relatively low muscle mass [41]. Overall, the prevalence of metabolic syndrome among adolescents with PH was within the range of 15–20%, in comparison with 0.2–2.8% depending on age, in the general paediatric populations of European countries (IDF definition) [42–45]. In the USA, using the definition formulated by Cook et al., metabolic syndrome was diagnosed in up to 8.6% of young people from the general population, more commonly among males [46].

### Hyperuricaemia and primary hypertension

Elevated serum uric acid levels do not constitute a criterion of metabolic syndrome, but are associated with metabolic syndrome abnormalities in both children and adults [32]. The tendency for elevated serum uric acid levels, even in the upper normal range (> 5.5 mg/dl), was found to be typical for adolescents with PH, and distinguished them from those with white-coat hypertension and secondary hypertension [47]. In a recent report from the SHIP-AHOY study, it was stated that the mean serum uric acid concentrations increased from 5.3 to 5.9 mg/dl with increasing blood pressure values from below the 80th to above the 90th percentile [28]. Moreover, Feig et al. reported that allopurinol used in adolescents with PH and serum uric acid levels above 6 mg/dl lowered both the level of uric acid and blood pressure [48].

### Oxidative stress

Oxidative stress (SOX) is a non-specific marker of metabolic abnormalities, which accompanies metabolic syndrome and is typical for visceral obesity. Elevated urinary isoprostane excretion has been found in obese children and adolescents and was associated with visceral obesity, but not with BMI and blood pressure [49]. Other studies which included hypertensive children demonstrated that the serum levels of asymmetric dimethylarginine and symmetric dimethylarginine were significantly increased in hypertensive children [50]. Although SOX is typical for obese children when compared with non-obese children, hypertensive children are exposed to greater SOX, irrespective of their BMI [36]. SOX markers also correlate with 24-h SBP. In a prospective study, it was observed that hypertensive children were exposed to greater SOX, and SOX markers correlated with left ventricular hypertrophy and the presence of metabolic syndrome [51].

### Accelerated biological maturation

It has been observed on a population level that accelerated biological development is associated with higher than average blood pressure, as confirmed in the National Health and Nutrition Examination Survey (NHANES) II and III, in which more advanced bone age in relation to chronological age was associated with higher blood pressure [52]. The association between accelerated maturation and visceral obesity, metabolic abnormalities, and elevated blood pressure was also demonstrated in other studies. In a prospective study conducted in Poland, earlier maturation was associated with higher BMI and higher BP in adulthood [53]. The same was observed in retrospective studies conducted in Iceland, which showed that rapid growth between 8 and 13 years of age was associated with elevated BP in adulthood and greater mortality and morbidity from CVD in adulthood [54, 55]. Similarly, the Fels Longitudinal Study showed that earlier growth spurt is associated with higher blood pressure, adiposity, and significant metabolic abnormalities, already in young adulthood [56]. In the Bogalusa Heart Study, it was found that early menarche was associated with fatness and increased risk of metabolic syndrome as well as PH in early adulthood (19–37 years) [57]. The same was also observed in the Cardiovascular Risk in Young Finns Study [58]. As early as 1980, in clinical observational studies, it was found that increased growth rate and more advanced bone age were associated with higher blood pressure and PH in adolescence [59]. In our study, we found that the difference between bone age and chronologic age in hypertensive children was 1.5 years, and accelerated biological development correlated with BP status ranging from normotension through prehypertension and ambulatory hypertension to severe ambulatory hypertension [60]. The mechanism of cause and effect relation between accelerated biological maturation and PH is not known. As suggested by Lever and Harrap, faster biological maturation is associated with greater exposure to growth factors, sex hormones and especially androgens, and visceral fat deposition [61]. Second, VAT is active hormonal tissue generating both androgens and corticosteroids, especially in women, which may accelerate biological maturation and elevate blood pressure [62–64].

### Immune abnormalities

There is an increasing amount of data, from both experimental and clinical studies, indicating that PH is associated with immune abnormalities and the activation of both innate and adaptive immunity. In children suffering from PH, the activation of the innate immune system is closely associated with the presence of metabolic syndrome, and high sensitivity C-reactive protein (hsCRP) levels correlate with a number of metabolic syndrome criteria [65]. Both SAT and VAT generate adipocytokines which modulate anti- and proinflammatory reactions. In children with PH, peripheral blood leukocytes express adiponectin receptors, and the expression is inversely correlated with the serum adiponectin levels irrespective of BMI; however, it correlates with the severity of hypertension—the more severe the hypertension, the greater the expression of adiponectin receptors and lower adiponectin concentrations [66]. Matrix metalloproteinases (MMPs) and their tissue inhibitors (TIMPs), which control extracellular matrix remodelling, are secreted by cells of the immune system. It was observed that the pattern of their secretion and gene expression was significantly disturbed in hypertensive children and was associated with VAT [67, 68]. Adolescents with PH also show subtle but significant alterations of adaptive immunity, such as alterations in the distribution of T cells, with more mature memory T cells and a lower percentage and number of regulatory T cells [69, 70]. According to recent findings, activation of both innate and adaptive immunity in PH leads to arterial wall remodelling and development of hypertensive target organ damage (TOD) and sustains hypertension [71].

### Increased sympathetic activity

Although there are no data concerning direct measurements of the activity of sympathetic nerves in children with PH, there is a large amount of data from clinical studies in which the activity of the sympathetic nervous system was assessed on the basis of heart rate, heart rate variability, hyperkinetic circulation, heart rate and blood pressure rhythms, and catecholamine concentrations [72]. As mentioned, increased sympathetic drive is associated with adiposity. In the Tecumseh study, conducted in the 1970s, it was observed that there was an interrelationship between blood pressure, the adrenergic drive, and adiposity: elevated blood pressure and heart rate at the age of 6 were associated with increased adiposity measures at the age of 22 [73]. Both blood pressure and adiposity measures were associated with the markers of adrenergic drive. Blood pressure rhythmicity was also significantly disturbed in children suffering from PH, and correlated with metabolic abnormalities typical of metabolic syndrome, especially with amount of VAT [74, 75]. It must be noted that in some studies, a significant increase of sympathetic activity, expressed as disturbed heart rate variability, was observed in hypertensive children and the effect was independent of adiposity [76]. However, in this study, the effect of VAT compartment was not analyzed.

## Metabolic syndrome, associated immune and developmental alterations, and hypertensive target organ damage

The above-described main abnormalities found in children with PH and typical of metabolic syndrome, immune activation, and sympathetic drive, are closely associated with TOD. It has been documented that the prevalence of left ventricular hypertrophy (LVH) correlates with the exposure to metabolic syndrome criteria, and severe LVH was found only in hypertensive patients who suffered from metabolic syndrome [42]. Similarly, subclinical hypertensive arterial injury, expressed as increased carotid intima-media thickness (cIMT), correlates with lower adiponectin concentrations and higher inflammatory activity [42, 65]. The same associations between LVH and arterial hypertensive injury, expressed as cIMT, pulse wave velocity (PWV), and central blood pressure, augmentation pressure, and index, were found with markers of SOX, disturbed distribution of memory T cells and regulatory T cells, adiponectin receptor expression on peripheral blood leukocytes, altered MMP/TIMP concentrations, and MMP/TIMP gene expression patterns [42, 51, 67–70, 77]. Recently, the usefulness of metabolic syndrome in terms of predicting TOD in comparison with the sum of its components has been questioned. An analysis of paediatric data indicated that metabolic syndrome diagnosed according to the IDF definition or the criteria by Cook et al. had similar values, as a sum of 3 components, in predicting the cIMT value in children. However, the sum of five components had a greater value in predicting cIMT in comparison with the diagnosis of metabolic syndrome based on 3 criteria [78].

## Concept of metabolically healthy and metabolically unhealthy obesity

The features of intermediate phenotype of PH are also typically seen in obese children and adolescents. They show altered body composition, features of immune system activation, accelerated biological maturation, and metabolic abnormalities typical of metabolic syndrome. A more detailed analysis indicates that in obesity, haemodynamic alterations, AH, and CVD risk depend on the distribution of adipose mass and the above-described metabolic abnormalities typical of metabolic syndrome. It led to the development of the concept of MHO and MUHO phenotypes. Recently, an operational definition of MHO in children was proposed; it defines MHO as a lack of metabolic and haemodynamic criteria of metabolic syndrome in obese children, even with increased WC [79]. The comparison of children and adolescents in relation to their anthropometric and metabolic phenotype showed that blood pressure was significantly higher in NWMU and MUHO compared with that in NWMH and MHO [80]. A more detailed analysis of body composition indicated that WC and the amount of VAT assessed by DXA were significantly higher in the case of NWMU and MUHO than in NWMH and MHO patients, respectively, and VAT amount determined metabolic abnormalities [80, 81]. Generally, children and adolescents with MUHO were older and their BMI was higher than in the case of MHO patients. It was also observed that puberty doubled the risk of switching from MHO to MUHO, which corresponds with the earlier description of the intermediate phenotype in adolescents with PH and the hypothesis of early vascular ageing in PH [82, 83]. A study conducted by Guzzetti el al. demonstrated a significant decline in the prevalence of MHO among obese children occurring with age—from 59.8% at the prepubertal stage to 30.8% at the pubertal stages 4–5 [84]. It was associated with increase in the prevalence of PH, from 5.9% at the prepubertal stage to 23.9% at the pubertal stages 4–5, and the increased prevalence of MUHO. Interestingly, even though the prevalence of metabolic abnormalities increased with age in both sexes, males more often than females showed pathological WC and PH (37.5 vs. 19.5%). Greater prevalence of PH among obese boys in comparison with obese girls was seen already at the prepubertal stage and amounted to 7.5 vs. 4.9%, respectively. The MUHO and NWMU phenotypes are determined by the same early life determinants as PH. More detailed analyses of young adults aged 31 showed that patients with MUHO and NWMU had lower birth weight than patients with MHO and NWMH [85].

The problem with the concept of MHO is whether it is truly benign or merely a transitory stage to MUHO. With age, there is a decrease in insulin sensitivity and so-called metabolic health. A recent analysis by Smith et al. indicated that after 4 to 20 years, MHO evolved to MUHO in 30 to 50% of patients [86]. Moreover, although the risk of CVD is lower in patients with MHO than in patients with MUHO, it is still higher than in NWMH subjects. It was observed that children with MHO had higher blood pressure than NWMH and NWMU children, and the blood pressure values correlated with both low adiponectin serum levels and higher leptin-to-adiponectin ratio [87]. In a prospective study, it was demonstrated that after 6 years of observation, at the age of 16, the risk of developing PH was significantly greater in children with MHO than in those who were NWMH (relative risk 5.42). Thus, the diagnosis of MHO means only that the CVD risk is lower than in the case of patients suffering from MUHO, but blood pressure and the risk of developing PH and metabolic abnormalities are still higher than in children with normal weight and normal body composition [84]. The distribution of adipose tissue plays a role in the development of metabolic and haemodynamic complications of obesity. According to the adipose tissue expandability hypothesis, increased amount of SAT protects against visceral fat deposition [85]. However, with time and increasing absolute volume of fat, this protective effect may decrease.

To conclude, the prevalence of PH among obese children is greater than in the general paediatric population, especially in obese patients suffering from MUHO. The prevalence of MUHO and PH among obese children increases with age. However, both non-obese children with PH and obese children show similar accompanying abnormalities. It indicates that the main pathophysiological mechanisms of PH and obesity-related hypertension are almost the same if not identical, and the treatment is based on the same principles. However, the risk of non-haemodynamic complications of obesity, such as non-alcoholic fatty liver disease and type 2 diabetes, is additionally greater in patients with MUHO phenotype. Polycystic ovary syndrome in viscerally obese girls, who show metabolic abnormalities, features of hyperandrogenism, and PH, constitutes a specific form of the MUHO phenotype [88].

## Principles of treatment

The treatment of PH and obesity-related hypertension depends on the stage of hypertension, comorbidities, and presence of TOD. The most important element of the treatment is non-pharmacological therapy, which is based on changing the lifestyle, modifying the diet, and taking up physical activity. The dietary modifications are the same as the modifications used in obesity treatment and in general are based on the reduction of caloric intake, simple carbohydrates, red meat, and salt. It is advised to consume more vegetables and lean, white meat instead of red meat. Three months of dietary treatment, based on the DASH diet applied in 57 adolescents suffering from prehypertension and PH, caused a greater decrease in SBP than a routine hospital-prepared diet [89]. However, due to the fact that altered body composition is one of the main features of both PH and obesity-related hypertension, physical activity constitutes a very important part of treatment. The paediatric guidelines of both the European Society of Hypertension and the American Academy of Paediatrics recommend at least 60 to 90 min of moderate-to-vigorous physical activity daily, both as a preventive measure and as a non-pharmacological treatment [90, 91]. The data from the European Youth Heart Study revealed that 116 min of daily physical activity in children aged 9 and at least 88 min in children aged 15 may prevent the clustering of cardiovascular risk factors including blood pressure elevation and the metabolic risk factors [92]. The current “2018 US Department of Health and Human Services’ Physical Activity Guidelines for Americans” recommend 60 min of moderate-to-vigorous physical activity daily plus at least 3 h of bone and muscle strengthening activity per week [93]. However, it is not only physical activity, but also improved fitness that matters. Cross-sectional paediatric studies indicate that there is a significant relationship between better fitness and favourable arterial phenotype [94]. There are significant differences between different forms of physical activity regarding energy expenditure expressed as metabolic equivalent units. The issue has been recently reviewed by Baker-Smith et al. [93]. A more detailed exercise prescription in patients with cardiovascular risk factors, including obese and hypertensive patients, has been recently published [95]. Non-pharmacological treatment based on physical activity is contraindicated in the case of some accompanying comorbidities, including structural heart disease, rhythm disturbances, myocarditis, pericarditis, and uncontrolled stage 2 or higher hypertension [93].

Although the effects of non-pharmacological treatment have been assessed in studies on children with obesity, type 2 diabetes, or non-alcoholic fatty liver disease, there are only a few published studies where the effects on blood pressure and/or regression of TOD were assessed. In an interventional, controlled study in prepubertal, obese, and hypertensive children (average age 9 years), 60 min of physical activity was applied 3 times a week for 3 months [96]. It was observed that after 3 months, the children from the intervention group had significantly lower blood pressure, lower prevalence of PH, decreased amount of abdominal and whole-body fat, and increased cardiorespiratory fitness. After 6 months, in addition to lower blood pressure, the patients who exercised also showed lower stiffness of the carotid artery and increased insulin sensitivity. In another study, Woo et al. compared the effects of diet alone and diet combined with physical activity in obese children aged 10 [97]. They observed that both diet and diet plus exercise led to an improvement in the endothelium-dependent dilation of the brachial artery; however, the effect of diet combined with physical activity was much better. Moreover, the continuation of training led to further improvement after 1 year. Importantly, detraining led to a decrease in the endothelial function as soon as after 6 weeks. The effects of non-pharmacological treatment combined with pharmacological therapy based on angiotensin-converting enzyme inhibitors (ACEi) or angiotensin receptor blockers (ARB) were assessed in a prospective, interventional study conducted on 86 adolescents with PH [43, 51, 77]. After 1 year of treatment, there was a significant decrease in blood pressure with normalization of blood pressure in 70% of patients, decrease in the prevalence of metabolic syndrome by 50%, decrease of SOX, immune activity, and regression of hypertensive TOD. The main determinants of hypertensive arteriopathy regression and normalization of the left ventricular geometry were a decrease in WC and increase in insulin sensitivity.

If applied, non-pharmacological treatment is very effective. The results of a meta-analysis of studies on adults showed that more intensive, supervised physical activity and more frequent visits and longer contact with health care professionals were associated with greater reduction of blood pressure [98]. From the practical point of view, it is not the selected form of physical activity that matters most, but rather patients’ compliance, their acceptance of non-pharmacological therapy, and physical activity corresponding to their preferences. It must be pointed out that non-pharmacological treatment when properly planned and used is much more effective and safer than pharmacological treatment. It is more effective because it directly affects the main pathophysiological abnormalities associated with PH such as the following: disturbed body composition, metabolic abnormalities, sympathetic activation, and probably immune abnormalities. It is safer because it does not lead to adverse drug reactions and complications related directly to drug action, such as with beta-adrenolytics. However, there are still challenges with implementing lifestyle changes. They depend on the attitudes of the physician, the patient, and the patient’s family. One of the most commonly encountered situations in our clinical practice as hypertension specialists is the recommendation to refrain of physical activity and exemption from physical classes given by family practitioners/general paediatricians to children with PH. The second big challenge in implementing lifestyle changes is patient adherence. It must be pointed out that in most cases, dietary changes must be implemented by the whole family, not only by the hypertensive child. The same is true with support given by family and friends, including schoolmates and teachers. Analysis of data provided by Williamson et al. shows that good effects of physical exercise are associated not only with supervised sessions of exercise, their intensity, and frequency but also with support given by nurses and physicians [98]. Last, but not least, parent education and SES of family are important.

Pharmacological treatment of PH is recommended in patients with stage 2 hypertension and TOD and/or in whom 6 to 12 months of non-pharmacological treatment failed [90, 91]. Due to the fact that the pathogenesis of both PH and obesity-related hypertension and the development of TOD are strictly associated with metabolic abnormalities, the choice of antihypertensive medications should be based on their metabolic effects. ACEi and ARBs have the most favourable metabolic profile; they increase peripheral blood flow and insulin sensitivity and are preferred as first-line medications [99–101]. The dihydropyridine calcium channel blockers are metabolically neutral, or may have a mild favourable metabolic effect, and can be introduced as the second medication or the first-line medication if ACEi/ARBs are contraindicated. In practice, the most common contraindication to ACEi/ARB is their use in sexually active women who do not use any form of contraception. Another contraindication to ACEi is atopy, angiooedema, and allergy to Hymenoptera insects. In these cases, ARBs seem to be safe. Although sodium restriction is effective in lowering blood pressure, thiazides/thiazide-like diuretics are not recommended in adolescents with PH and metabolic syndrome, because of their potential to further aggravate IR and hyperuricaemia. Similarly, beta-adrenolytics aggravate IR. A new option is nebivolol, which is a new beta 1 receptor blocker with vasodilating properties and the potential to induce nitric oxide generation through endothelial cells [102]. A comparison with metoprolol indicated that nebivolol significantly increased insulin sensitivity [103]. Although there are no data concerning the use of nebivolol in children, it has been registered for use in adults and may be used in older adolescents.

As mentioned previously, allopurinol lowers blood pressure and may be recommended in subjects with accompanying hyperuricaemia. Metformin, which increases insulin sensitivity, is often used as an additional drug in the case of IR and may promote weight loss [104, 105].

## Conclusions

PH and obesity-related hypertension share similar intermediate phenotype with metabolic abnormalities typical of metabolic syndrome and is a complex neuro-immuno-metabolic disease. Disturbed body composition and visceral obesity play a crucial role in the development of metabolic abnormalities and TOD, and the risk of TOD increases with the number of metabolic risk factors. A decrease in VAT and increase in lean body mass are the main determinants of blood pressure reduction, TOD regression, and normalization of metabolic abnormalities. For this reason, non-pharmacological therapy based on modifications of diet and lifestyle, including increased physical activity, is the mainstay of the treatment. During the selection of pharmacological treatment, the physician should take into account not only the antihypertensive efficacy of medications, but also their metabolic effects. In some cases, additional drugs may be used to treat accompanying metabolic disturbances.

### Key summary points

- Primary hypertension and obesity-related hypertension is a complex neuro-immuno-metabolic disease complicated by arterial hypertension.
- Disturbed body composition and visceral fat play a key role in the pathogenesis of primary hypertension and obesity-related hypertension.
- Non-pharmacological treatment based on diet, lifestyle, and physical activity modifications is the basis of treatment.
- Pharmacological therapy of primary hypertension and obesity-related hypertension should consider adverse metabolic effects of some antihypertensive medications.

### Multiple choice questions (answers follow the references)

1. The prevalence of primary hypertension and of obesity-related hypertension among children and adolescents

- Is the same in boys and girls
- Is higher in girls in comparison with boys
- Is higher in boys than in girls and increases with growth spurt
- Is higher in prepubertal girls than in pubertal boys

2. The phenotypic features of primary hypertension include

- Slower biological maturation, metabolic abnormalities typical of metabolic syndrome, increased parasympathetic activity, disturbed body composition
- Accelerated biological maturation, hyperoestrogenism, metabolic abnormalities typical of metabolic syndrome, disturbed body composition
- Accelerated biological maturation, metabolic abnormalities typical of metabolic syndrome, tendency to lower serum uric acid levels, disturbed body composition
- Accelerated biological maturation, metabolic abnormalities typical of metabolic syndrome, tendency to elevated serum uric acid, disturbed body composition, increased sympathetic activity

3. Serum uric acid levels in adolescents with primary hypertension are:

- Normal and do not differ from normotensive individuals
- Lower than in normotensive children
- Elevated in secondary hypertension and lowered in primary hypertension
- Tend to be higher in primary hypertension and treatment with allopurinol lowered both serum uric levels and blood pressure

4. Pharmacological antihypertensive treatment in adolescents with primary hypertension and metabolic syndrome should be based on:

- ACEi/ARBs and, when contraindicated, dihydropyridine calcium channel blockers
- Beta-adrenolytics
- Beta-adrenolytics and thiazides/thiazide-like diuretics
- Centrally acting agents

5. Non-pharmacological treatment of primary hypertension and obesity-related hypertension

- Is the most important part of treatment and should be started in all patients with stage 1 hypertension and should accompany pharmacological therapy in patients with stage 2 hypertension
- Should be used only in patients with stage 1 hypertension
- Dietary treatment alone has the same efficacy as diet plus physical activity
- Physical activity should be increased to 3 days a week for 60 min

1. c; 2. d; 3. d; 4. a; 5. a
